# Supplementary material for: Establishment of an In Vitro Model to Study Viral Infections of the Fish Intestinal Epithelium
Source: Cells. 2023 Jun 1;12(11):1531. doi: 10.3390/cells12111531 (PMC10252704; doi:10.3390/cells12111531)
Supplement: Supplementary file 1 [file cells-12-01531-s001.zip › cells-2371528-supplementary/Figure S2.pdf]

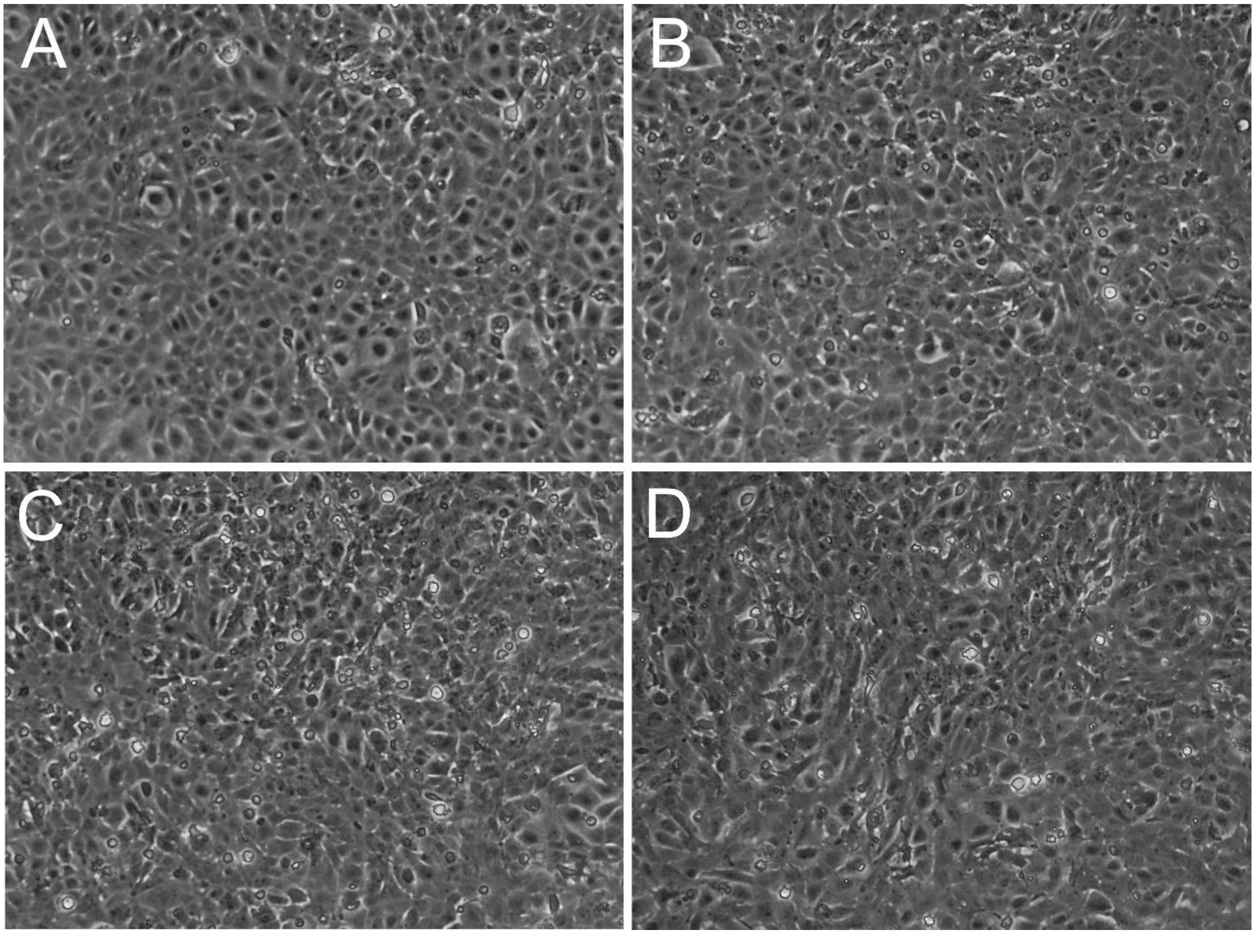

Figure S2. Phase contrast microscopical images of RTgutGC cells inoculated with SAV3 at different MOI at 5 days post infection. A) Uninfected RTgutGC cells. B, C and D) Cells inoculated with SAV3 at MOI 0.1. 1 and 10, respectively.
