# Supplementary material for: Establishment of an In Vitro Model to Study Viral Infections of the Fish Intestinal Epithelium
Source: Cells. 2023 Jun 1;12(11):1531. doi: 10.3390/cells12111531 (PMC10252704; doi:10.3390/cells12111531)
Supplement: Supplementary file 1 [file cells-12-01531-s001.zip › cells-2371528-supplementary/Table S1.pdf]

Table S1. Primers used for qPCR

Gene name	Acronym	Primer sequences 5'-3'	Accession no.
Infectious pancreatic necrosis virus	*ipnv*	F-CAACAGGGTTCGACAAACCATAC<br>R-TTGACGATGTCGGCGTTTC	AY379740.1
Salmonid alpha virus E2	*sav3*	F-CAGTGAAATTCGATAAGAAGTGCAA<br>R-TGGGAGTCGCTGGTAAAGGT	LN625236.1
Infectious salmonid anemia virus (seg 8)	*isav*	F-GGCTATCTACCATGAACGAAT<br>R-GCCAAGTGTAAGTAGCACTCC	HQ259678.1
Interferon alpha	*ifn-a*	F-TGGGAGGAGATATCACAAAGC<br>R-TCCCAGGTGACAGATTTCAT	NM_001123570.1
Protein kinase K	*pkr*	F-TGAACACAGCCAGAAGAACAA<br>R-GACTACCGCCACATAACTCCA	EF523422.1
Myxovirus resistance protein 1	*mx-1*	F-TGCAACCACAGAGGCTTTGAA<br>R-GGTTGGTCAGGATGCCTAAT	U66475
Interferon regulatory factor 9	*irf9*	F-AAGGAGGAGGAGGTTGTGGT<br>R-CGAACTGGTCTTGTTGGATG	NM_001173719.1
Tumor necrosis factor alpha	*tnf-α*	F-AGGTTGGCTATGGAGGCTGT<br>R-TCTGCTTCAATGTATGGTGGG	NM_001124357
Beta-actin	*actb*	F-CAAAGCCAACAGGGAGAAGATGA<br>R-ACCGGAGTCCATGACGATAC	NM_001124235
